# Deep subwavelength fourfold rotationally symmetric split-ring-resonator metamaterials for highly sensitive and robust biosensing platform

**DOI:** 10.1038/srep02437

**Published:** 2013-08-14

**Authors:** Landobasa Y. M. Tobing, Liliana Tjahjana, Dao Hua Zhang, Qing Zhang, Qihua Xiong

**Affiliations:** 1 OPTIMUS, Photonics Centre of Excellence, School of Electrical and Electronic Engineering, Nanyang Technological University, Singapore 639798; 2 School of Physical and Mathematical Sciences, Nanyang Technological University, Singapore 637371

## Abstract

Metamaterials provide a good platform for biochemical sensing due to its strong field localization at nanoscale. In this work, we show that electric and magnetic resonant modes in split-ring-resonator (SRR) can be efficiently excited under unpolarized light illumination when the SRRs are arranged in fourfold rotationally symmetric lattice configuration. The fabrication and characterization of deep subwavelength (~λ/15) gold-based SRR structures with resonator size as small as ~ 60 nm are reported with magnetic resonances in Vis-NIR spectrum range. The feasibility for sensing is demonstrated with refractive index sensitivity as high as ~ 636 nm/RIU.

Metamaterials are artificially structured media consisting of array of metallic split-ring resonators (SRR) that can be designed to give rise to novel electromagnetic properties such as negative magnetic permeability and negative refractive index^1^. Many efforts have been dedicated to realize metamaterials in all electromagnetic spectrum^1^,^2^,^3^,^4^ due to its potential applications in super-lensing, invisibility cloaking, molecular spectroscopy, and ultrasensitive biochemical sensing^5^,^6^,^7^,^8^,^9^,^10^. The prerequisite for building a metamaterial is that the size (*s*) and lattice constant (*a*) of the constituting metallic resonators should be much smaller than the operating wavelength (λ). However, the realization of a metamaterial is increasingly difficult towards shorter wavelength operation. To date^10^,^11^,^12^,^13^, the smallest fabricated SRR has the size of ~ 100 nm, exhibiting distinct magnetic resonance at infrared wavelength. For achieving magnetic resonance in visible spectrum (400 nm < λ < 800 nm), one can estimate that the size of SRRs should be smaller than 100 nm, indicating the need of a large scale sub-30-nm patterning capability.

Having magnetic resonance in the visible spectrum range is highly desirable since most experiments on biochemical sensing and molecular spectroscopy employ coherent/broadband visible light source. Compared to integrated optics based sensors^14^, metamaterials (or metallic resonators in general) exhibit much higher sensitivity due to intense electromagnetic field localization within nanoscale gaps. Furthermore, metamaterial-based sensing is carried out in a microscopic setting where transmission and reflection spectra are locally extracted and analyzed, in contrast to the integrated optics sensors which require optical alignment with submicron scale precision. However, broadband light sources are mostly unpolarized, making the excitation of SRR modes inefficient due to their strong polarization dependence. Excitation of electric and magnetic modes in SRRs using randomly polarized light is particularly desirable in the context of having a simple and robust sensing platform where the sensing is implemented in microscopic setting without the need for polarization control of input/output light signals.

In this article, we report fourfold rotationally symmetric SRR lattices that exhibit significantly enhanced electric and magnetic resonances under unpolarized light illumination. We demonstrate the fabrication and characterization of deep-subwavelength SRR lattices that have magnetic resonances within the visible spectrum, with the smallest SRR of ~ 60 nm size and ~ 20 nm feature width. The capability of such lattices for biochemical sensing is also studied, and the refractive index sensitivity as high as ~ 636 nm/RIU is observed for fundamental magnetic resonance in the near infrared wavelength range.

## Results

**Figure 1:**
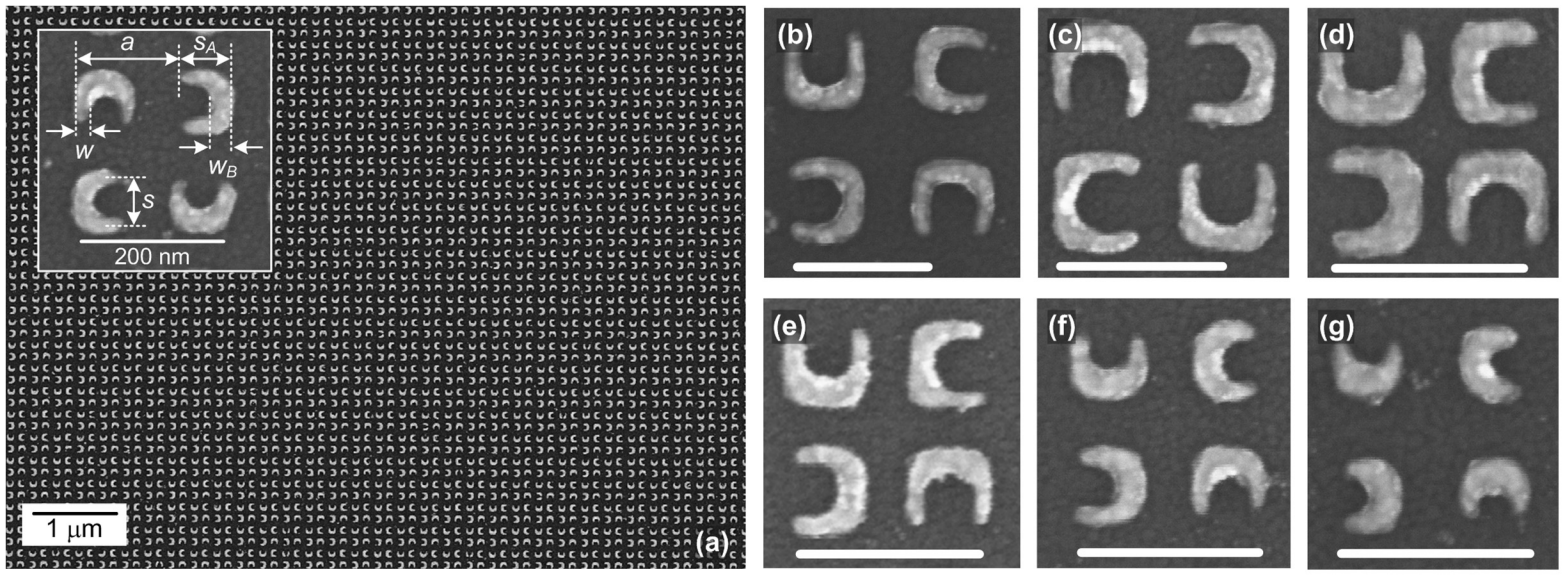
Large-scale arrays of fourfold rotationally symmetric split ring resonator lattices. (a) *s* = 70 nm, *w* ~ 20 nm, *w*_B_ ~ 35 nm, *s*_A_ ~ 80 nm, and *a* = 140 nm. (b) *s* = 100 nm, *a* = 200 nm, (c) *s* = 100 nm, *a* = 160 nm, (d) *s* = 90 nm, *a* = 140 nm, (e) *s* = 80 nm, *a* = 140 nm, (f) *s* = 70 nm, *a* = 140 nm, (g) *s* = 60 nm, *a* = 140 nm. For (b)–(g), *w* ~ 20 nm and the scale bar is 200 nm.

The fabrication of sub-100 nm sized SRR does not only require tens of nanometer features, but also good pattern fidelity. The latter requires fabrication process with steep contrast curve (more details are given in the supplementary information). The SRR lattices in this work were fabricated by a robust electron beam lithography (EBL) process we developed recently^15^,^16^, which has very high contrast (γ ~ 25) and is capable of sub-15-nm patterning at low exposure dose (<100 μC/cm^2^). In the typical square lattice configuration, both magnetic and electric modes cannot be excited effectively under randomly polarized light because all the SRRs are oriented in the same direction and thus only support one polarization. However, by reorienting the resonators in particular directions, it is possible to have a situation in which such polarization dependence is not perceived by the incoming randomly polarized light. In this work, we explore the use fourfold rotationally symmetric SRR lattice configuration (corresponding to point group *C*_4_) for this purpose, as shown in Fig. 1.

Each SRR sample is 100 μm × 100 μm in footprint, on either ITO coated glass (for transmission mode) or silicon substrate (for reflection mode). Gold is chosen as the constituting metal. The SRRs have a line width of w ~ 20 nm and thickness in the range of *h* ~ 24–28 nm (thicker than the skin depth of gold^17^). The nominal size (*s*) is varied from *s* = 60 nm to *s* = 100 nm, and the lattice constant (*a*) is varied from *a* = 140 nm to *a* = 200 nm [Fig. 1(b)–(g)]. The highest achievable density based on our process is 90-nm sized SRR at 140-nm lattice constant (Fig. 1d), which corresponds to only ~ 30 nm gap separation between two adjacent SRRs. Good pattern fidelity can be observed for SRR size as small as 60 nm, which to the best of our knowledge, marks the smallest lithographically patterned SRR so far.

**Figure 2:**
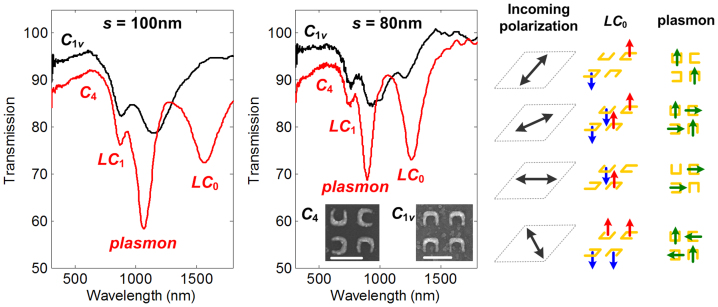
Enhanced excitation in fourfold rotationally symmetric lattice. Transmission spectra of SRRs (*a* = 200 nm) configured as square lattices (*C*_1*v*_), and fourfold rotationally symmetric lattices (*C*_4_) for *s* = 80 nm and *s* = 100 nm, respectively. The scale bar is 200 nm. The interaction between unpolarized light with *C*_4_ configuration is illustrated, with the arrows representing the 4 possible light polarizations, the magnetic moment (for *LC*_0_ mode) and dipole mode (for plasmon mode).

The transmission spectra of the metamaterial structures, with nominal sizes of 80–100 nm and a lattice constant of *a* = 200 nm, arranged in square lattice (*C*_1*v*_) and in fourfold rotationally symmetric (*C*_4_) lattice for the unpolarized light illumination are shown in Fig. 2. The magnetic (*LC*_0,1_) and electric (plasmon) resonances are denoted accordingly in the figure, while the general characteristics of magnetic and electric modes are briefly discussed in the *Methods* section. For the same density and size, it can clearly be seen that the *C*_4_ case exhibits much higher resonance contrast compared to that of *C*_1*v*_. The interaction between SRR and randomly polarized (RP) light is so enhanced that *LC*_0_ mode that cannot be observed in *C*_1*v*_ lattice (for *s* = 100 nm) can now be seen with clear resonance dip in the *C*_4_ lattice. The same is observed for 80-nm sized SRR where both *LC*_0_ and plasmon modes have markedly higher contrast compared to those in *C*_1v_ case.

The enhancement (η) is deduced by comparing the resonance contrasts of C_4_ and C_1v_ lattices, i.e., η = ΔT(C_4_)/ΔT(*C*_1*v*_). The resonance contrast (ΔT) is defined as ΔT = *T*_peak_–*T*_dip_, where *T*_dip_ is the transmission at the resonance dip while *T*_peak_ is the transmission at the nearer (and smaller) peak. For *s* = 80 nm, the resonance contrast of *LC*_0_ modes (ΔT_LC0_) are ΔT_LC0_ ~ 1.24% (for *C*_1*v*_) and ΔT_LC0_ ~ 18% (for *C*_4_), indicating an order of magnitude enhancement (η_LC0_ ~ 14×). On the other hand, the resonance contrast for plasmon modes (ΔT_plasmon_) are ΔT_plasmon_ ~ 7% (for *C*_1*v*_) and ΔT_plasmon_ ~ 18.35% (for *C*_4_), corresponding to η_plasmon_ ~ 2.6× enhancement. It is interesting to see that the enhancement for *LC*_0_ mode is higher than that for plasmon mode. We believe that such enhancement in *LC*_0_ and plasmon modes is mainly because the *C*_4_ lattice appears invariant from four different angles, thereby translating to reduced polarization dependence to the incident RP light. This is illustrated in the right panel of Fig. 2, with 4 possible polarizations of RP light for the sake of simplicity. For *LC*_0_ mode, one can see that the magnetic dipoles generated in the SRRs within one unit cell are in opposite directions with each other, causing zero net magnetic dipole. This remains true for all polarization directions. On the other hand, the case for plasmon mode is rather different where the induced dipoles in one unit cell tend to follow the polarizations of the incoming light. Thus, by comparison alone, one can see that the polarization dependence in *C*_4_ lattice for both *LC*_0_ and plasmon modes is much weaker than those in *C*_1*v*_ that prefer only one polarization direction. By further comparing *LC*_0_ and plasmon modes in *C*_4_ lattice configuration, it can be seen that the *LC*_0_ displays weaker polarization dependence, which seems to explain why the enhancement of *LC*_0_ mode is higher than that of the plasmon mode.

**Figure 3:**
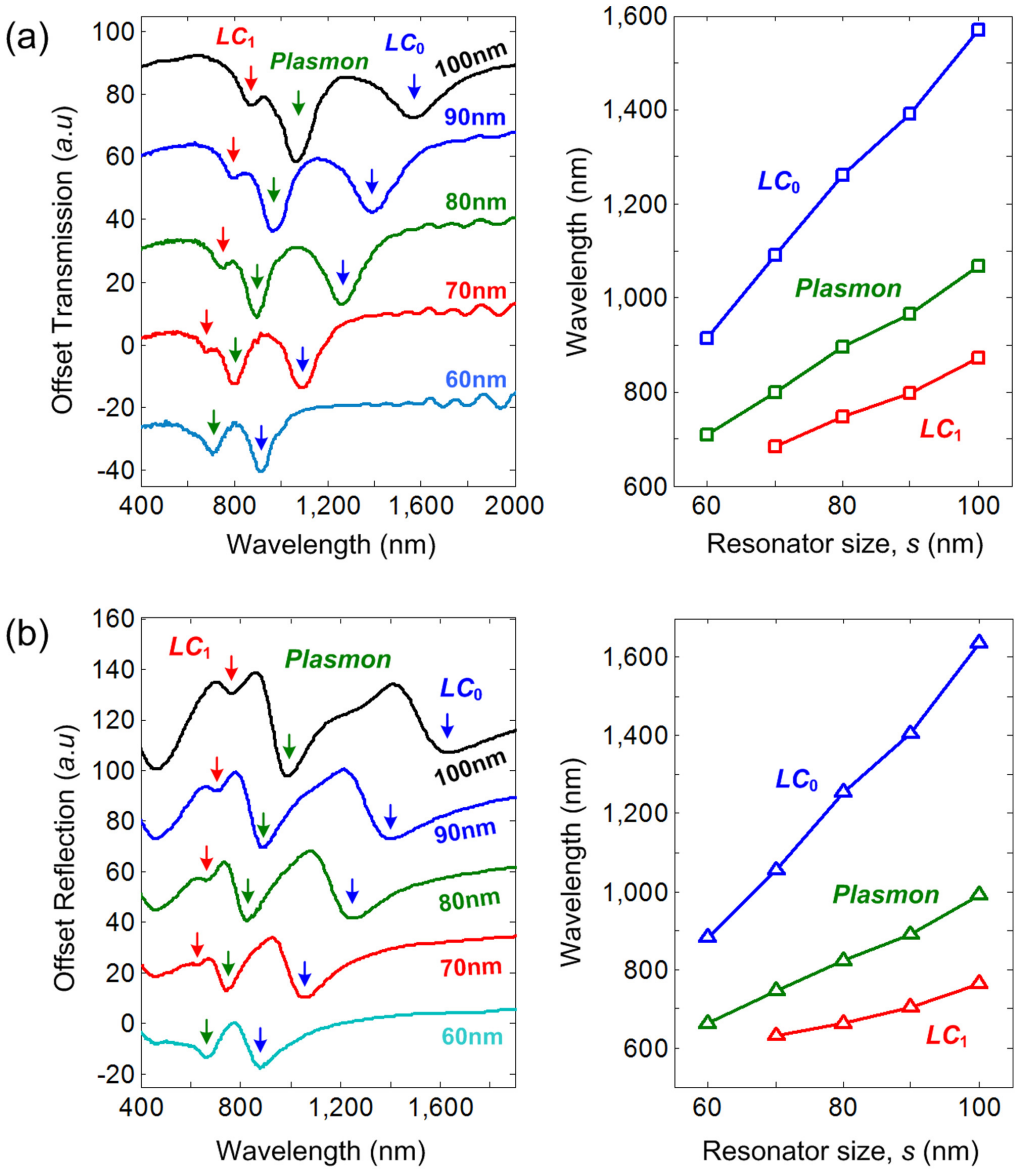
Magnetic and electric resonances of Au-based split ring resonator. (a) On ITO-coated glass substrate and (b) Si substrate.

In Fig. 3, we present the electric and magnetic resonant modes as a function of resonator size for *C*_4_ SRR lattices, where the *LC*_0_, plasmon, and *LC*_1_ modes are denoted by blue, green, and red arrows, respectively. Here, the lattice constant is fixed to 200 nm for both ITO-coated glass and silicon substrate. Generally the resonance wavelengths are in good agreement with those demonstrated by other groups, although comparatively longer. This is attributed to smaller feature size (*w* ~ 20 nm) and thickness (*h* < 30 nm) compared to other SRR structures that normally have the width and metal thickness in ~ 30–40 nm range^10^,^11^,^12^. Finite difference time domain (FDTD) simulations were also performed to investigate the effect of feature size and thickness (more details in supplementary information), which confirms that the SRR exhibits longer resonance wavelength when the width (thickness) is narrower (smaller)^18^,^19^,^20^.

The linear correlation between the resonance wavelength and the resonator size is clearly observed, as expected from the generic *LC*-circuit model^21^. The *LC*_0_ resonance shifts from ~ 1569 nm (*s* = 100 nm) to ~ 914 nm (*s* = 60 nm) for ITO-coated glass substrate, and from ~ 1635 nm (*s* = 100 nm) to ~ 884 nm (*s* = 60 nm) for silicon substrate. This shows that the SRRs in this work already enter deep subwavelength range, which generally have the size of ~ λ/15 and are separated by ~ λ/8 lattice constant. As the resonance wavelength decreases further, the kinetic inductance starts to set in and the optical loss increases^22^, progressively lowering the resonance contrast. The resonant mode eventually diminishes when the resonator size goes below 70 nm at which the resonance wavelength is shorter than ~ 700 nm. The shortest measured fundamental magnetic resonance wavelength of Au-based SRR is λ_LC0_ ~ 914 nm (for ITO-coated glass) and λ_LC0_ ~ 884 nm (for silicon substrate), while the shortest measured higher order magnetic resonance is λ_LC1_ ~ 682 nm (for ITO-coated glass) and λ_LC1_ ~ 630 nm (for silicon substrate). We also observed that the resonance wavelengths are shorter for silicon case than for ITO case, which might be attributed to the presence of ITO that is known to induce red shift^18^.

**Figure 4:**
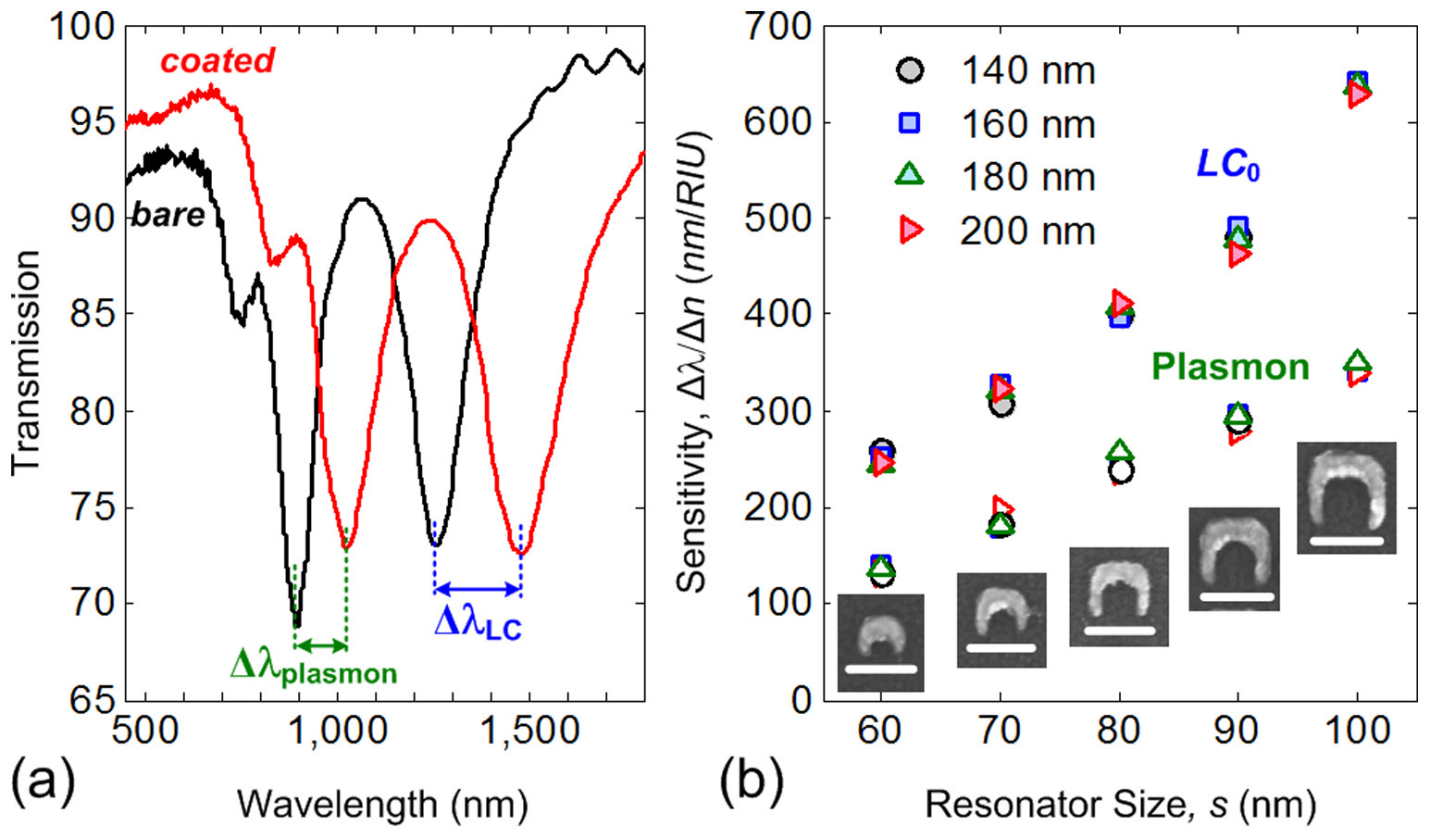
The effect of size on refractive index sensitivity. (a) The transmission spectra of 80-nm sized fourfold rotationally symmetric SRR lattices with and without coating. (b) Measured sensitivity (γ) of SRR with different size and density. The scale bars in all insets are 100 nm.

In order to explore sensing capability of this fourfold rotationally symmetric SRR lattice structures, the samples were coated by ~ 100 nm thick ZEP positive tone resist (*n*_ZEP_ ~ 1.54 for Vis-IR range), and followed by measuring refractive index sensitivity (Γ) that is defined as the wavelength shift (Δλ) over the change of refractive index unit (RIU), i.e., Γ = Δλ/Δ*n*. Here, ~ 100-nm thick dielectric layer represents the size of a virus, which is in 20–300 nm range. Figure 4a shows the transmission spectra of the 80-nm sized SRRs (on ITO-coated glass) before and after coating of the ZEP resist. Note that the resonance contrast of electric and magnetic resonant modes remains unaffected after coating. The measured wavelength shifts are ~ 218 nm (for *LC*_0_ mode), ~ 132 nm (for plasmon mode) and ~ 93 nm (for *LC*_1_ mode). The dependence of sensitivity on resonator size and density at *LC*_0_ (filled markers) and plasmon (hollow markers) resonances are shown in Fig. 4b, where sensitivity as high as Γ ~ 636 nm/RIU (Γ ~ 339 nm/RIU) is observed for 100-nm sized SRR structure for *LC*_0_ mode (plasmon mode). The sensitivity is expected to be even higher when the resonators are functionalized to attract specific molecules, for example the use of covalent thiol chemistry^23^ to bind specific protein such as bovine serum albumin (BSA).

**Figure 5:**
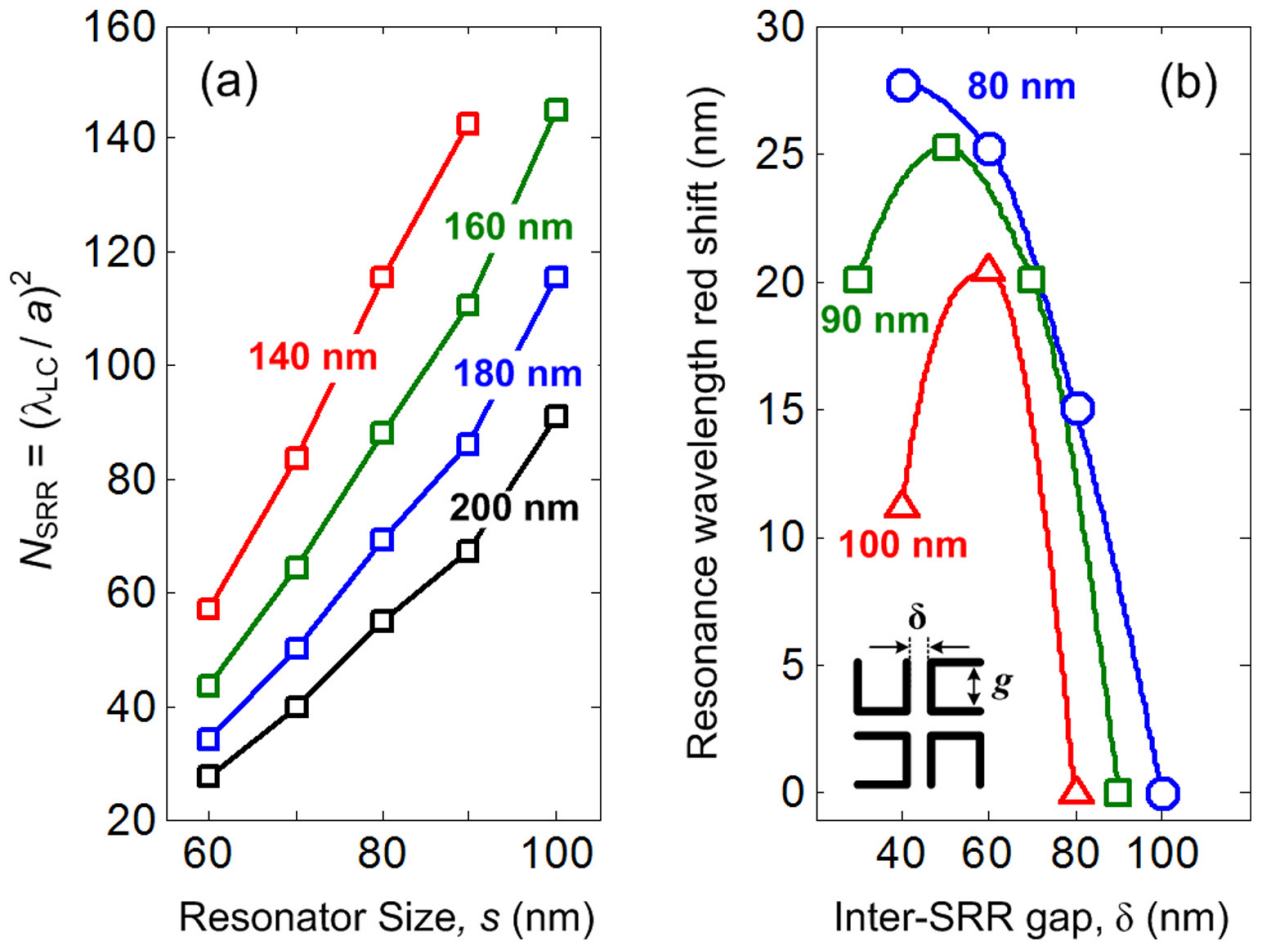
The role of inter-resonator coupling. (a) Estimated number of resonators per unit photon for different SRR sizes and densities, where a single photon is assumed to cover an area of ~ λ^2^. (b) The wavelength red shift of *LC*_0_ mode as a function of inter-SRR gap for different resonator size.

While SRR density does not significantly affect sensitivity, one can see that the sensitivity of *LC*_0_ mode decreases from Γ ~ 636 nm/RIU to Γ ~ 250 nm/RIU as the resonator size decreases from *s* = 100 nm to *s* = 60 nm. This can be explained from a simple *LC*-circuit reasoning. The dependency of *LC*-resonance on cladding permittivity (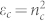) and resonator size (*s*) can be expressed as 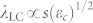, which leads to the conclusion that the sensitivity is also dependent on resonator size, since 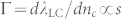. One way to interpret this dependency is that the sensitivity depends on the “dipole density” that is perceived by the incoming light. By assuming that a single photon covers an approximate area of *A*_photon_ ~ λ^2^, the effective number of resonators interacting with a single photon (*N*_SRR_) at lattice constant *a* can be estimated as *N*_SRR_ ~ (λ_LC_/*a*)^2^, where *λ*_LC_ is the *LC*_0_ resonance wavelength of the coated SRR structure. Since 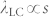, one can thus arrive at 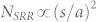, indicating that the perceived dipole density increases when the resonator size increases. Thus, this results in higher sensitivity, as shown in Fig. 5a which plots *N*_SRR_ as a function of resonator size and lattice constant. This also suggests that the sensitivity is not affected by lattice configuration. The measured spectral shifts for 80-nm sized SRR in *C*_1v_ configuration are ~ 184 nm (for plasmon mode) and ~ 170 nm (for LC_0_ mode), which corresponds to Γ ~ 341 nm/RIU (plasmon mode) and Γ ~ 315 nm/RIU (*LC*_0_ mode). On the other hand, the resonance shift and sensitivity of 100-nm sized SRR (for plasmon mode) are ~ 216 nm and Γ ~ 401 nm/RIU, respectively. The slight differences between the sensitivities of *C*_4_ and *C*_1v_ may be attributed to the differences in SRR thickness which leads to different resonance wavelengths, and eventually to the change of the sensitivity (see Fig. 4). The other factor is the rather ambiguous measurement of *C*_1v_ SRRs due to low resonance contrast (See Fig. S5).

The effect of inter-resonator coupling is presented in Fig. 5b which shows the wavelength shift as a function of inter-SRR gap. The inter-SRR gap is δ = *a* – (*s* + *w*), while the wavelength shift is measured from *LC*_0_ resonance wavelength at *a*_0_ = 200 nm, i.e., 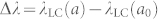. One can see that the inter-SRR coupling results in red shift of the resonance wavelength, indicating that the inter-SRR coupling is dominated by longitudinal coupling of electrical dipoles^24^. This is described by a quasi-static interaction energy 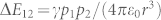 with γ as the interaction index, i.e., γ = 1 (for transversal coupling) and γ = −2 (for longitudinal coupling)^25^. Note that the magnitude of longitudinal coupling is ~ 2× stronger than that of transversal coupling for the same distance. In addition, since γ < 0 for longitudinal coupling, the excitation of parallel dipoles (*p*_1_*p*_2_ > 0) gives rise to negative interaction energy, leading to a red shift in resonance wavelength. It should also be noted that the transversal coupling between magnetic dipoles also exist. However, the strength is weaker than that between the electrical dipoles, particularly because the magnetic dipole strength is known to decrease towards shorter wavelength^26^. Finally, the effect of strong coupling is also investigated where the inter-SRR gap is smaller than the SRR gap (δ < *g*). By substituting SRR gap as *g* = *s* – *w*, and inter-SRR gap as δ = *a* – *s* – *w*, the condition for strong coupling can be expressed as *a* < 2 *s*. From Fig. 5b, one can see that the red shift initially increases with the decreasing gap separation. However, as it enters strong coupling region (δ < *g*), the red shift saturates and starts to decrease as the gap separation is further decreased. We attribute this to the inter-SRR capacitance effect.

## Discussion

We have successfully demonstrated fabrication and characterization of gold-based split ring resonator structures that have magnetic resonances within visible wavelength spectrum. Split-ring resonators as small as ~ 60 nm size and ~ 20 nm feature width have been successfully fabricated with good pattern fidelity. Magnetic and electric resonances of Au-SRR lattices have been characterized with the shortest fundamental magnetic resonance at ~ 914 nm (on ITO coated glass) and ~ 884 nm (on silicon); and higher order magnetic resonance at ~ 682 nm (on ITO coated glass) and ~ 630 nm (on silicon). Further resonance wavelength reduction is possible for metals with lower optical loss and higher plasma frequency such as silver (Ag) and aluminum (Al). We have also demonstrated that the excitation of magnetic and electric modes under randomly polarized light can be enhanced by simply arranging the SRRs into fourfold rotationally symmetric lattice configuration. This is especially important for realizing robust, simple, and cost-effective biochemical sensing platform. By comparing the resonance contrast of the fourfold rotational symmetric lattice (*C*_4_) and square lattice (*C*_1*v*_) configurations, we achieved ~ 14 × enhancement of fundamental magnetic resonant mode and ~ 2.6× enhancement for plasmon mode. Using fundamental magnetic resonance for sensing, a sensitivity of ~ 636 nm/RIU has been demonstrated around near infrared wavelength range, which is much higher than those based on integrated optics devices, in addition to the simpler optical setup that does not require polarization control and high precision optical alignment. Finally, the effect of inter-SRR coupling is investigated and strong coupling is observed when the inter-SRR gap is smaller than SRR gap.

## Methods

### SRR fabrication

The split ring resonators (SRR) were fabricated by electron beam lithography (EBL) on ITO coated glass (for transmission mode) or silicon substrate (for reflection mode). To realize large scale metamaterial structures at short writing time, we employed ultrahigh contrast EBL process based on sonicated cold development that is able to achieve ultrahigh resolution patterns at low exposure dose^15^. The e-beam patterning was carried out at 20 keV beam energy, using ~ 20 pA beam current. The SRR structures were written as a series of single pixel lines at ~ 400 pC/cm line exposure dose. Each metamaterial sample with specific resonator size and density was realized on 100 μm × 100 μm footprint. The resonator size is varied from 60 nm to 100 nm, while the lattice constant is varied from 140 nm to 200 nm, giving 20 variations in total. The writing time for each metamaterial footprint (including the markers) is about 4 minutes. Then ~ 24–28 nm thick gold film was deposited by e-beam evaporation technique (Edwards 306) at 0.05 nm/s deposition rate, followed by lift-off in dimethyl acetamide (ZDMAC) solution for ~ 10 minutes at 70°C. Due to different process batches, the SRR thicknesses are slightly different, where (for *C*_1v_ lattice) the thickness of gold is 24 nm (where 3-nm thick titanium was used as adhesion layer), and (for *C*_4_ lattice) 28 nm where no adhesion layer is used. The adhesion layer is known to impart red shift to resonance wavelength, but does not significantly affect the resonance contrast (as long as it is within 5 nm thickness)^17^,^18^. In addition, it was found that ~ 4 nm difference in Au thickness only gives marginal impact to the resonance wavelength (as evidenced in Fig. 2).

### SRR characterization

We used CRAIC micro-spectrophotometer to locally extract transmission and reflection spectra, which are collected by 36 × objective lens at 25 × 25 μm sampling area using unpolarized broadband source (UV-Vis-NIR). The transmission (reflection) was normalized with the background spectrum of ITO glass (silicon) substrate. Although *LC*_1_ mode is not excited under normal incidence, we nevertheless still observe *LC*_1_ mode in our transmission/reflection spectrum. This is attributed to the numerical aperture of our objective lens (NA = 0.4), which also collects off-normal incidence signals. Using a simple expression for numerical aperture, i.e., 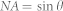, the acceptance angle of the objective lens is θ = ± 23.5°, which is sufficiently oblique for *LC*_1_ mode excitation (although not optimum because the off-normal incidence signal is much weaker than the normal incidence signals). This is consistent with the fact that LC_1_ mode has much lower resonance contrast than do *LC*_0_ and plasmon modes, and the fact that no enhancement was observed for LC_1_ mode in the *C*_4_ lattice configuration.

### Finite difference time domain calculation

The finite difference time domain simulation was done by commercial 3D optiFDTD software, where a Gaussian pulse input plane wave (of *x*-polarization) was launched into a split ring resonator structure with 30 nm thickness, 20 nm side arm width, and 35 nm bottom arm width. The resonator size was varied from 60 nm to 100 nm. The material property of gold was taken from the software database, and the grid size for all simulations was fixed to 5 nm. The magnetic resonances (*LC*_0,1_) were identified by the peaks in the Discrete Fourier Transform (DFT) spectrum of *H*_z_-field.

### Mode characteristics of split ring resonator

**Figure 6:**
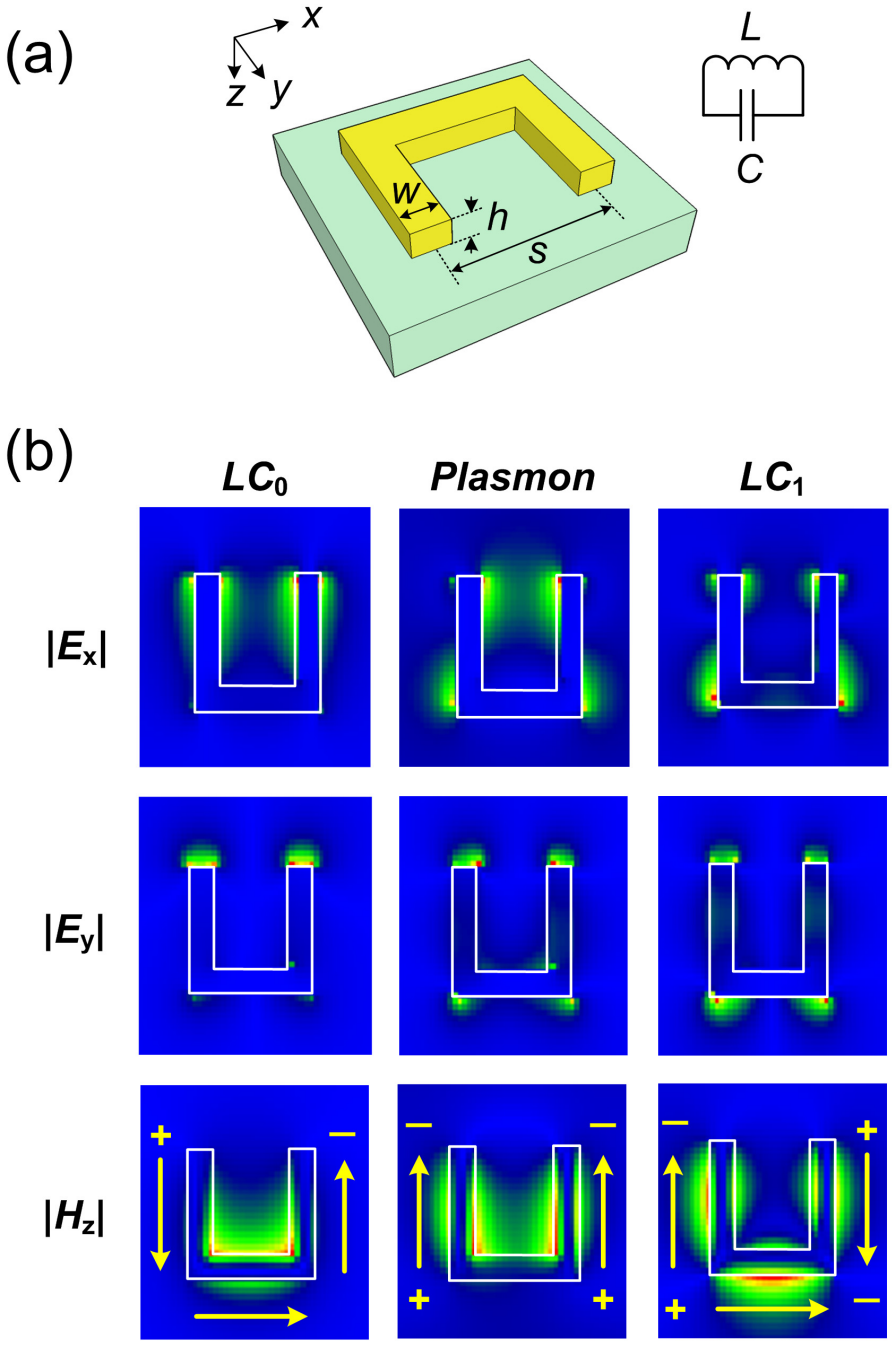
Mode characteristics of SRR. (a) Schematic of SRR. (b) Electromagnetic field profiles of magnetic and electric modes.

As shown in Fig. 6a, a split ring resonator is a *U*-shaped metallic structure that resembles an *LC*-oscillator circuit, where the gap separation between the SRR side arms and the circumferential length of the SRR constitute the capacitance (*C*) and inductance (*L*), respectively. Generally, a SRR accommodates both the electric and magnetic modes. The former is often referred to as plasmon mode, while the latter is often known as *LC*-resonance. The electromagnetic field profiles of these modes are shown in Fig. 6b. The lowest order mode is the fundamental magnetic resonance (*LC*_0_). In this case, a strong horizontal dipole is excited between the SRR tips (as shown in the concentrated *E*_x_ and *E*_y_ fields), leading to a circulating current that produces magnetic field perpendicular to the SRR plane (as shown in the localized *H*_z_-field). The resulting permeability is thus negative (μ < 0), and the SRR behaves as a magnetic dipole^3^. The oscillation frequency of the fundamental magnetic resonance is characteristically the same as that of a *LC*-oscillator, i.e., *ω*_LC_ = (*LC*)^−1/2^. Since horizontal dipole mode is the prerequisite for the formation of the circulating current, it follows that *LC*_0_ mode can only be excited by horizontally polarized light. Meanwhile, the other two higher order modes (plasmon and *LC*_1_) can be understood in the perspective of symmetric and anti-symmetric mode hybridization resulting from mutual coupling between two dipole modes from two SRR side arms. The symmetric case corresponds to the plasmon mode, in which the dipoles are parallel with each other. Due to the same charge polarity induced on the side arms, there is no induced dipole along the bottom arm. This is verified by the fact that *H*_z_-field is only localized around the side arms. Strong localization of *E*_y_-field shows that plasmon mode is dominated by vertical dipoles, indicating that plasmon mode can only be excited when the incident light is vertically polarized. On the other hand, the anti-symmetric case corresponds to *LC*_1_ mode where the vertical dipoles are anti-parallel with each other, and horizontal dipole is induced as a result of opposite charge polarity in the bottom arm. The magnetic field is rather delocalized towards the outer side of the SRR, showing that this is higher order magnetic resonance. Due to its anti-symmetricity, exciting *LC*_1_ mode requires the excitation of vertical dipoles at different time, thus making the excitation of LC_1_ mode difficult, unless it is done under oblique incidence^3^.

## Author Contributions

D.H.Z. initiated and supervised the project. L.Y.M.T. conceived the idea and fabricated the SRR structures. Q.Z. and Q.X. facilitated the micro-spectrophotometer measurements. L.Y.M.T., L.T. and Q.Z. performed the SRR characterizations. L.Y.M.T. and L.T. performed the FDTD analysis. L.Y.M.T. wrote the manuscript, while L.T. and D.H.Z. did the manuscript editing. All the authors read and approve the manuscript.

## Supplementary Material

Supplementary Information Supplementary Information
