# Supplementary material for: Deep subwavelength fourfold rotationally symmetric split-ring-resonator metamaterials for highly sensitive and robust biosensing platform
Source: Sci Rep. 2013 Aug 14;3:2437. doi: 10.1038/srep02437 (PMC3743072; doi:10.1038/srep02437)
Supplement: Supplementary Information [file srep02437-s1.pdf]

Deep subwavelength fourfold rotationally symmetric split-ring-resonator metamaterials for highly sensitive and robust biosensing platform

Landobasa Y. M. Tobing[1]*, Liliana Tjahjana[1], Dao Hua Zhang[1]*, Qing Zhang[2], Qihua Xiong[2]

[1]School of Electrical and Electronic Engineering, Nanyang Technological University, Singapore, 639798.
[2]School of Physical and Mathematical Sciences, Nanyang Technological University, Singapore, 639798.

Supplementary Information

Sonicated cold development EBL process

The contrast curve of sonicated cold development is shown in Fig. S1 [denoted as (3)], which shows ~5x increase in the contrast compared to standard process [denoted as (1)]. [15, 16]. The contrast is defined as the inverse of the slope around transitional dose region, $\gamma=\log_{10}(D_{100}/D_0)$, where D_0 (D_{100}) denotes onset dose (clearing dose).

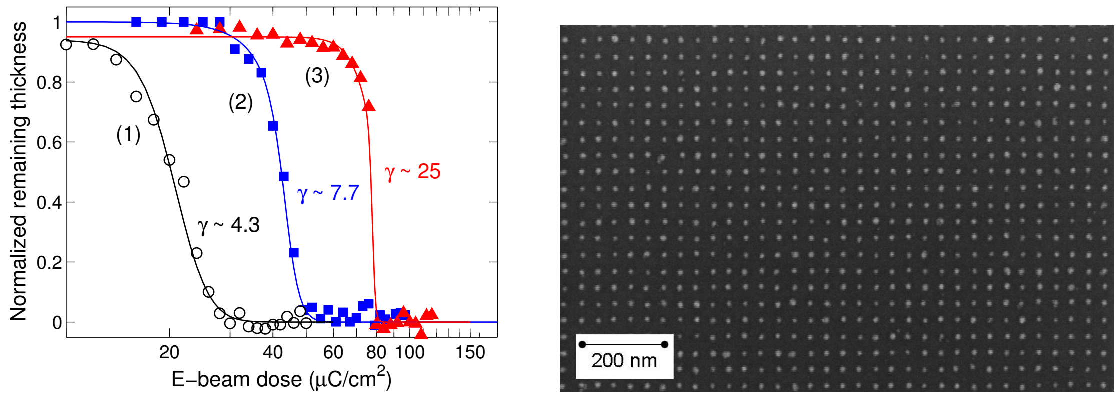

Fig. S1. (Left) Contrast curve of different EBL processes: (1) Standard development, (2) cold development, and (3) sonicated cold development process. (Right) The example of using sonicated cold development for nanometal patterning: 40-nm pitch sub-15-nm sized gold dots after lift-off, which is patterned by 20kV EBL process (20pA current and ~50μC/cm² exposure dose).

FDTD calculation of Split Ring Resonator

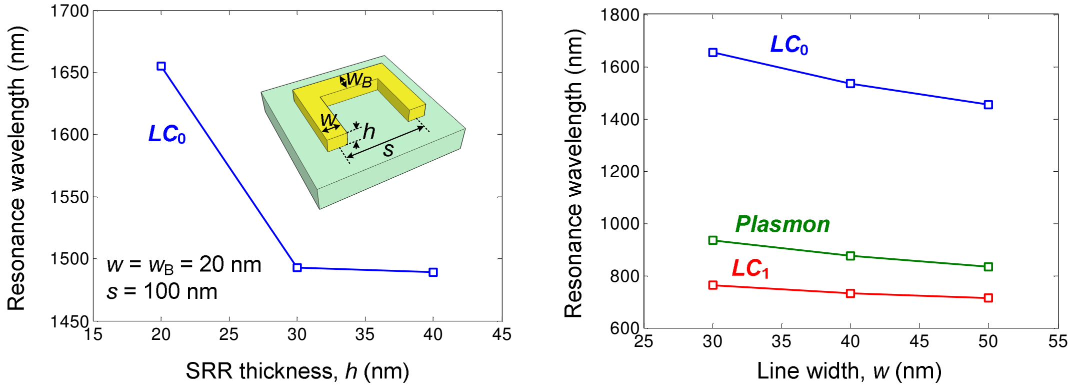

Fig. S2. (Left) The dependence of LC_0 resonance wavelength on SRR thickness. (Right) The effect of feature width to SRR resonance modes (the thickness and size are fixed to 30 nm and 100 nm, respectively).

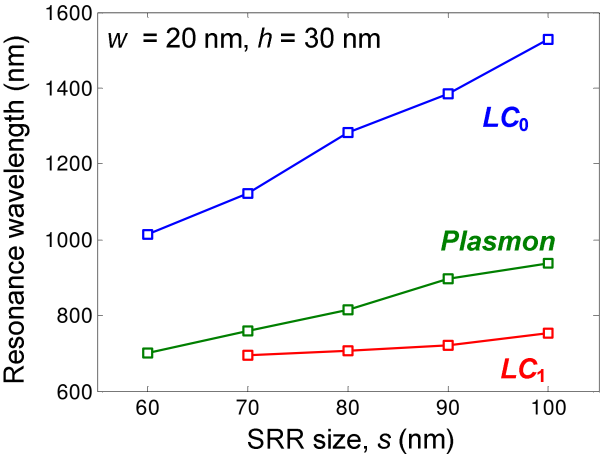

Fig. S3. The SRR resonant modes for different resonator sizes. Here, the bottom arm width (w_B) for each resonator size is chosen to be as close as possible to SEM measurements. For s = 90-100 nm, w_B = 30 nm, while for s = 60-80 nm, w_B = 35 nm.

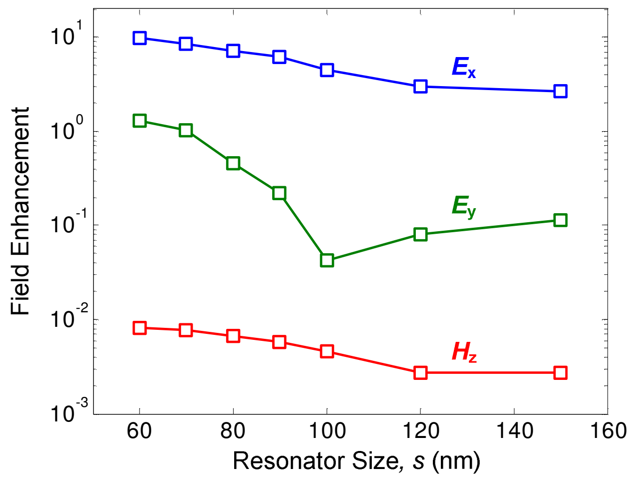

Fig. S4. Calculated field enhancement of E_x, E_y, and H_z fields at LC_0 resonance for different resonator sizes. The enhancement is defined as the ratio between the field at the center of SRR gap and the field of input light.

<u>Measurement of refractive index sensitivity of C_{1v} lattices</u>

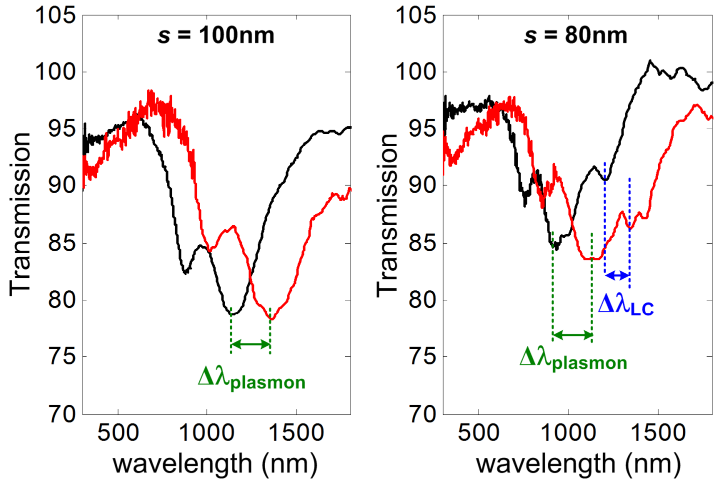

Fig. S5. The transmission of C_{1v} SRR lattice under different cladding for s = 100 nm and s = 80 nm.
